# Debates in Allergy Medicine: Does oral immunotherapy shorten the duration of milk and egg allergy? The pro argument

**DOI:** 10.1186/s40413-018-0191-6

**Published:** 2018-06-15

**Authors:** Valentina Pecora, Rocco Luigi Valluzzi, Maurizio Mennini, Vincenzo Fierro, Lamia Dahdah

**Affiliations:** Division of Allergy, IRCCS Ospedale Pediatrico Bambino Gesù, Piazza Sant’Onofrio, 4, 00165 Rome, Italy

**Keywords:** Cow’s milk, Desensitization, Hen’s egg, Oral immunotherapy, Oral tolerance, Sustained unresponsiveness

## Abstract

The development of oral tolerance or food allergy is an active process, related to dynamic interactions between host immune cells, microbiome, dietary factors, and food allergens. Oral tolerance is the default immune response in the gut. A food allergy occurs when this process fails and a pathologic Th2 response is activated. Oral food immunotherapy (OIT) aims to restore immune tolerance in food-allergic individuals. The stimulation of Tregs production seems to represent a crucial step in inducing long-term tolerance, but other mechanisms (e.g., the suppression of mast cell and basophil reactivity, changes in allergen-specific cells with regulatory markers) are involved. Several studies reported the efficacy of OIT in terms of "sustained unresponsiveness" (SU), an operational definition of immune tolerance. In successfully treated subjects, the ability to pass an oral food challenge 2 to 8 weeks after stopping the food allergen exposure seems to be conditioned by the treatment starting age, frequency, amount or type of food consumed, and by the duration of the maintenance phase. Based on the available data, the percentage of milk- and egg-allergic subjects achieving sustained unresponsiveness after an OIT ranges from 21% to 58,3%. A comprehensive understanding of mechanisms underlying the induction of oral tolerance with OIT, or natural tolerance to food allergens in healthy individuals, could potentially lead to advances in development of better treatment options for food allergic patients.

## Background

Despite increasing knowledge in oral tolerance, the current standard of care in treating food allergy according to the international guidelines is still a strict elimination diet [1–6]. However, the dietary approach has several limitations. First, the risk for severe systemic reactions due to the presence of hidden allergens [7, 8] in food products in spite of best efforts at strictly avoiding food allergens. Second, avoidance diets may be associated to the risk of nutritional deficiencies and impaired growth especially if the food/s involved represent fundamental component of the conventional diet (such as cow’s milk or hen’s egg) [9]. Third, inadvertent exposure to food ingredients is an everyday risk. Therefore, considering the increasing prevalence of food allergy [10, 11] with a significant impact on the public health in industrialized countries [12], attempts to modify the immune response to foods are a required choice, particularly in severe food allergies [13]. Oral immunotherapy (OIT) aims to do so through food exposure.

The first report of successful desensitization performed in a hen’s egg allergic patient dates back to 1908 [14], and until the end of the 1990s only a few sporadic cases were reported [15, 16]. The use of subcutaneous route was related to high-risk of severe systemic reactions [17, 18] and was quickly abandoned. Starting from the end of the twentieth Century, an increasing number of OIT studies was reported in the literature. In addition to case reports [19, 20], clinical trials on OIT as an effective treatment for food allergy began to be published [21–24]. A hundred years after the first report, international scientific societies became interested in OIT.

With the resulting exponential increase in the number of clinical trials published, metanalyses became possible [25–29]. Their current evidence suggests a proved efficacy in short-term tolerance, while information on long-term outcomes is limited and mostly focused on milk OIT. The long-term follow-up studies [30–33] have proposed to evaluate only the regular intake of the incriminated food, sometimes reporting adverse reactions occurred during the follow-up period. Side effects commonly reported in the literature are the main weakness of this treatment, which is still not recommended in the routine clinical practice. Generally, most reactions arising from clinical trials are mild and limited to the oropharynx resolving without intervention or with antihistamine alone. However, systemic or severe reactions do not seem unlikely and are most frequent during the build-up phase commonly conducted under physician supervision.

### Oral tolerance to food protein in the gut

The gastrointestinal tract is the major route of exposure to food allergens and the largest reservoir of immune cells in the body. Intestinal commensal bacteria induce protective and regulatory responses that maintain host-microbial mutualism, and the mucosal immune system plays a crucial role protecting the gastrointestinal tract from invading pathogens and keeping the commensal microbiota compartmentalized. The epithelial cells, responsible for separating the mucosal immune system from the gut lumen, secrete a number of factors that contribute to barrier function, including mucins, antimicrobial peptides, and trefoil factors. This type of cells also transport antibodies, particularly IgA, into the intestinal lumen where these antibodies can contribute to barrier function by excluding the uptake of antigens or microbes [34]. The resident immune cells, located inside the matrix of the Peyer’s patches, include CD4^+^ and CD8^+^ T effector and regulatory T cells (T^regs^), B cells, macrophages and dendritic cells. The latter in particular are critical for maintaining immune homeostasis within the gut. Their major functions concern the processing and the presentation of antigens, a critical step in the activation of T cells. In detail, CD103^+^ dendritic cells in the mesenteric lymph nodes express high levels of the enzyme retinal dehydrogenase 2 (RALDH2), which converts retinal to retinoic acid promoting gut-homing activity and development of Tregs from naïve T cells as well as secretion of transforming growth factor β (TGF-β) [35, 36].

Gut-associated intestinal lymphoid tissue discriminates between potentially harmful pathogens and non-harmful antigens. Therefore, it is possible to observe an activation of a protective immune response or an ‘off’ state of T cell due to a functionally inactivation of lymphocyte following an antigen encounter, such as food or commensal bacteria [34].

The intestinal microbiota varies between individuals, and plays key roles in defense against pathogens as well as food digestion and nutrition. In case of dietary changes, a modification in bacterial metabolites (such as short-chain fatty acids that derive from fermentation of dietary fibers) is observed, with repercussions on mucosal integrity and inflammasome activation [37]. The inflammasome pathway and production of the cytokine interleukin (IL)-18 are critical for intestinal homeostasis and epithelial integrity by ensuring repair and cell survival under stress conditions [38, 39].

### Immunomodulation during a specific food allergen immunotherapy

The goal of food immunotherapy (oral, sublingual or epicutaneous) is to modify the immune response towards food protein antigens. Many studies report suppression of mast cell and basophil reactivity, a reduction of allergen-specific IgE and a simultaneous increase of allergen-specific IgG4 antibodies [40, 41]. At the same time, the interest of researchers was focused on Tregs, and specifically on two different populations: CD4 + CD25+ forkhead box P3 (Foxp3)^+^ Treg cells and Th3 cells. The inhibitory cytokine TGF-β is responsible for the mechanism of suppression provided by Th3 cells expressing a late-stage Treg activation marker, latency associated peptide (LAP), which forms a complex with TGF-β [42, 43]. Based on the recent evidences [41], Foxp3+ Tregs were induced by the three treatment routes but in particular by epicutaneous immunotherapy (EPIT). LAP+ T^reg^ levels increase in EPIT and OIT, whereas IL-10^+^ cells are induced by sublingual immunotherapy (SLIT). The suppressive activity of EPIT-induced T^regs^ required cytotoxic T-lymphocyte antigen 4 (CTLA-4), whereas SLIT is strictly dependent on IL-10 and OIT acted through both mechanisms. IL-10 represents a key cytokine inhibiting INF-γ and IL-2 secretion by Th1 cells and IL-4/IL-5 production by Th2 cells. The stimulation of T^reg^ production seems to represent a crucial step in inducing long-term tolerance. A boosting of antigen-specific serum IgA level was observed in a mouse model of food OIT [44]. In this case the neutralization by allergen-specific IgA would demonstrate a protective role. In addition, according to the murine model the OIT protection would be localized to the gastrointestinal tract with significant downregulation of gastrointestinal gene expression [44].

### Could OIT be conceived of as a disease-modifying treatment?

Until a few years ago, the possibility that OIT could be able to modify the natural history of food allergy was not expected. Many studies indicated that the maintenance of tolerance status obtained with the OIT required constant exposure to the food allergen [23, 45–47]. In 2012 the term “sustained unresponsiveness” (SU) was introduced for the first time [48], describing the ability of a food allergic subject, successfully desensitized with OIT, to pass an oral food challenge (OFC) conducted generally 28 weeks after stopping the food allergen exposure. Patients getting SU from their OIT will be allowed to introduce a previously allergenic food into their diet ad libitum, as happens to subjects who spontaneously acquire a clinical tolerance.

During the last 5 years, several clinical studies have been proposed to demonstrate the achievement of a SU in food allergic patients. Currently available data concern patients with cow’s milk, hen’s egg and peanut allergy. Considering that children allergic to milk and egg are most likely going to outgrow spontaneously their food allergies, all available data must be critically reviewed. In this regard, the age of enrollment should not be underestimated. All clinical trials [36, 49–54] published on SU achieved after an OIT with cow’s milk and hen’s egg are expected to enroll food allergic subjects aged over 5 years (Table 1). Different dosing schedules and varying durations in terms of maintenance phase and food avoidance period also make comparison between trials difficult. Based on the available data, the percentage of milk- and egg-allergic subjects achieved SU after an OIT ranges from 21% to 58,3% in a few years.

**Table 1:** Characteristics and results of hen’s egg and cow’s milk OIT RCTs

Egg	Burks et al. (2012)	Escudero et al. (2015)	Yanagida et al. (2016)	Jones et al. (2016)
Study design	RCT double blinded	RCT, not blinded	RCT, not blinded	RCT, follow-up
Age range (years)	5–11 (median age: 7 ys)	5–17 (median age: 8 ys)	≥5	5–11 (median age: 7 ys)
Number of patients (active group)	40	30	21	40
Number of patients (control group)	15	31	12	15
Withdrew from therapy (active group)	5	2	5	5
Withdrew from therapy (control group)	2	0	0	2
OIT duration	22 months	3 months	10 weeks	48 months
Maximum tolerated dose	2 g	one undercooked egg every 2 days	62–194 mg	2 g
DBPCFC after OIT in placebo group	At month 10 (5 g) 100% positive	Not performed	Not performed	Not performed
DBPCFC after OIT in active group	At month 10 22 negative (55%) (*P* < 0.001) At month 22 (10 g) 30 negative (75%) (*P* < 0.001)	Not performed	Not performed	Not performed
Time of elimination diet (weeks)	4–6	4	2	4–6
DBPCFC after food avoidance (cumulative maximum dose)	At month 24 (10 g) 11 negative	At month 4 (3,6 g) 1 negative in CG 11 negative in AG	At week 12 (3 g) 0 negative in CG 7 negative in AG	At month 36 18 negative At month 48 20 negative
Sustained unresponsiveness (%)	28 (P = 0.03)	37	33,3 (*P* = 0.032)	45% at year 3 50% at year 4
Milk	Yanagida et al. (2015)		Wood et al. (2016)	Takahashi et al. (2016)
Study design	RCT, not blinded		RCT, double blinded	RCT, not blinded
Age range (years)	≥5		7–35 AG median 11.7 years CG median 9.5 years	5–17 AG median 9 years CG median 7 years
Number of patients (active group)	12		28 (OIT plus omalizumab)	31
Number of patients (placebo group)	25		29(OIT plus placebo)	17
Withdrew from therapy (active group)	0		2	0 at year 1 11 at year 4
Withdrew from therapy (placebo group)	0		5	0
OIT duration	12 months		30 months	4 years
Maximum tolerated dose	3 ml		3.8 g	200 ml
DBPCFC after OIT in placebo group	4 negative (3 ml)		20 negative (10 g)	0 negative (80 ml)
DBPCFC after OIT in active group	9 negative (3 ml)		24 negative (10 g)	14 negative (80 ml)
Time of elimination diet (weeks)	2		8	2
DBPCFC after food avoidance (cumulative maximum dose)	At month 12.5 (3 ml) 4 negative in CG 7 negative in AG	At month 12.5 (25 ml) 0 negative in CG 4 negative in AG	At month 32 (10 g) 10 negative in CG 13 negative in AG	At year 1 (80 ml) 7 negative/31 At year 2 (80 ml) 14 negative/30
Sustained unresponsiveness (%)	58.3% *P* = 0.018	33.3% *P* = 0.007	35.7% in CG 48.1% in AG	At year 1 21% At year 2 47% *P* = 0.008

#### Egg

Burks and colleagues [48, 51] published their experience with OIT in egg allergic individuals by analyzing the results obtained at 2 and 4 years from the beginning of the research protocol. The goal was to desensitize the subjects to 2 g of egg-white powder, achieved by just under 50% (18/40) of subjects randomized to the active procedure group within the first 10 months. At 10 months and 22 months, all participants underwent an OFC consisting of 5 g and 10 g (cumulative dose) of egg-white powder respectively. At 22 months, 30 of 40 children (75%) in the OIT group successfully passed the challenge, discontinued OIT and avoided all egg consumption for 4 to 6 weeks. At 24 months, these children underwent an OFC with 10 g of egg-white powder to test for sustained unresponsiveness and 11 (27.5%) successfully passed the challenge (*P* = 0.03, as compared with placebo) with the resulting instruction to add egg to their diet ad libitum without specific recommendation on frequency, amount, or type of egg product. Considering the immune markers measured, small wheal diameters on skin-prick testing and increases in egg-specific IgG4 antibody levels were associated with passing the oral food challenge at 24 months. At a later point of time, the authors evaluated the efficacy and safety of egg OIT in the same participants treated up to 4 years [51]. Long-term follow-up questionnaires were administered after study conclusion (LFQ-1) and 1 year later (LFQ-2) to assess possible effects of the lifestyle on the outcomes of the study. At Years 3 and 4, all subjects treated with egg OIT underwent a 10 g (cumulative dose) OFC to egg white powder to assess desensitization. Those who passed the desensitization OFC discontinued OIT dosing for 4–6 weeks and had a second OFC (10 g as a cumulative dose), to assess for SU. At the fourth year of treatment, the percentage of patients who achieved SU rose to 50% (20/40). During both LFQ periods, the egg OIT–SU group showed a greater consumption of unbaked and baked egg in terms of frequency and amount compared to egg OIT-desensitized group. At year 4, subjects achieving SU had higher IgG4 values (*p* = 0.001) and lower egg skin prick test scores (*p* = 0.0002) over time and a lower median baseline ratio of egg-specific IgE to total IgE (1.1% vs. 2.7%, *p* = 0.04).

Recently, a 71% (15/21) SU to egg was reported 2 weeks after the discontinuation of a low-dose (1/32 egg) OIT, carried out for 12 months [50].

The efficacy of a short-course egg OIT to induce SU was also reported. Thirty-seven per cent of patients (11/30) passed an OFC performed at 4 months after a 30-day avoidance period [49]. These tolerance rates clearly exceed those expected from the natural history of egg allergy resolution. If these data will be confirmed, OIT should be considered a disease-modifying treatment in egg allergy.

#### Milk

A milk OIT, supported by the simultaneous use of omalizumab, was also reported to be associated with SU [53]. At month-28, omalizumab was discontinued and subjects passing an OFC continued OIT for 8 weeks, after which OIT was discontinued with re-challenge at month-32. SU was demonstrated in 13/27 (48.1%) of the active group. Afterwards, the authors sought to investigate mechanisms by which omalizumab modulates immunity in the context of OIT and to identify baseline biomarkers that predict subgroups of patients most likely to benefit from omalizumab [55]. A reduction of milk-induced basophil CD63^+^ expression was observed in omalizumab- and placebo-treated subjects. However, IgE dependent histamine release increased in washed cell preparations only from omalizumab-treated subjects. Baseline basophil CD63^+^ expression was strongly associated with the occurrence of symptoms during OIT. The degree of suppression in milk-induced CD63^+^ expression at months 28 and 32 was associated with the likelihood of passing an OFC at these visits, suggesting that inhibition of basophil reactivity might be central to the underlying mechanisms responsible for desensitization to milk. The combination of baseline basophil and serologic biomarkers allowed to define a subset of patients in which adjunctive therapy with omalizumab was associated with attainment of SU and a reduction in adverse reactions. Neither omalizumab- nor placebo-treated subjects exhibited a significant increase in the percentage of casein-specific Treg cells over the course of treatment.

The duration of maintenance phase appears to have a decisive influence on the achievement of SU in cow’s milk allergic subjects. To this end, a Japanese study demonstrated that, 2 years after the start of OIT, the rate of 2-weeks-SU in the active group significantly increased compared with the rates at 1 year (*P* = 0.008) [54].

There are many considerations to be made regarding the factors that might affect the achievement of a SU in food allergic subjects after an OIT. First, the age bias could represent a decisive variable and future studies should investigate whether treatment outcomes regarding desensitization or SU are influenced by OIT’s starting age. Second, the analysis of microbiome of food allergic subjects before and after OIT could provide useful information regarding the achievement of desensitization or SU [56]. Third, clinical tolerance induced by food immunotherapy is associated with changes in basophils, IgG_4_, allergen-specific Th2 cells, and allergen-specific cells with regulatory markers. The identification of significant changes from the baseline, correlated with SU, would be helpful to provide the necessary dietary information to patients. Unlike SU, the state of desensitization requires to continue a regular allergen intake indispensable to maintain the established tolerance. Forth, the food habits in terms of frequency, amount, or type of food product consumed (unbaked and baked) seem to directly influence the achievement of SU. Fifth, long-term follow-up studies on OIT will allow to obtain a global view with the consequence of identifying possible factors likely to predispose food allergic subjects to achievement SU.

## Conclusion

Despite a growing knowledge about the pathophysiologic mechanisms underlying allergic diseases, immune responses associated with tolerance still need investigation. Oral tolerance represents an active regulatory immune response. The mechanisms inducing oral tolerance are manifold and involve allergen-specific Treg cells generated by mucosal DC, intestinal mucins and cytokines coming from epithelial cells and innate lymphoid cells. Gut-associated intestinal lymphoid tissue discriminates between potentially harmful pathogens and non-harmful antigens, with a consequent functional inactivation of lymphocyte following ad antigen encounter (such as food or commensal bacteria). In addition, integrity of mucosal epithelial barrier and intestinal homeostasis are influenced by the inflammasome pathway and production of IL-18 [34, 35]. As for humoral mechanisms, the detection of allergen-specific IgG_4_ is especially associated with a clinical tolerance to foods. However, it is not clear if they represent an active mechanism of immune tolerance or a mere consequence of food exposure in subjects consuming allergenic foods.

Important assessments to be consider before starting an OIT include the type of offending food/s and the age of allergic subjects. Indeed, at least 80% of milk- and egg-allergic children are expected to achieve spontaneous clinical tolerance by the school age, whereas the percentage falls to 10–20% in the case of peanut- or tree nut–allergic subjects [57, 58]. For this reason, the OIT’s starting age is crucial to achieve reliable results especially in the case of milk or egg allergic patients.

The spontaneous resolution of food allergy in children is associated with an increased frequency of peripheral blood CD4^+^ CD25^+^ Tregs after an OFC and a reduced proliferation of food allergen specific T cells [59, 60]. The depletion of CD4^+^ CD25^+^ T^regs^ restores the in vitro proliferative response in food allergen tolerant individuals [53].

The literature data certainly support the hypothesis that the OIT is able to accelerate the resolution of food allergy. Indeed, this type of treatment aims to re-introduce safely the offending food into the diet in a relatively short time. The OIT is associated with a suppression of mast cell and basophil reactivity, with a consequent reduction of allergen-specific IgE and simultaneous increase of allergen-specific IgG4 antibodies. Subjects successfully treated with OIT showed changes in allergen-specific cells with regulatory markers, in particular Foxp3^+^ and LAP^+^ Tregs, which seem to play a central role in inducing long-term tolerance. The lack of acquisition regarding the SU in all treated patients underlies significant differences in individual immune response. In this context, emphasis should be placed on a more comprehensive understanding of mechanisms underlying the induction of oral tolerance with immunotherapy or natural tolerance to food allergens in healthy individuals, to enable the development of better treatment options for food allergic patients.

## Abbreviations

CTLA-4: Cytotoxic T-lymphocyte antigen 4
EPIT: Epicutaneous immunotherapy
Foxp3: Forkhead box P3
IL: Interleukin
LAP: Latency associated peptide
OFC: Oral food challenge
OIT: Oral immunotherapy
SLIT: Sublingual immunotherapy
SU: Sustained unresponsiveness
TGF-β: Transforming growth factor beta
T^regs^: Regulatory T cells

## References

[1] Boyce JA (2010). Guidelines for the diagnosis and management of food allergy in the United States: report of the NIAID-sponsored expert panel. J Allergy Clin Immunol.

[2] Fiocchi A, Brozek J, Schunemann HJ, Bahna SL, von Berg A, Beyer K (2010). World allergy organization (WAO) diagnosis and rationale for action against Cow’s milk allergy (DRACMA) guidelines. WAO J.

[3] Muraro A, Werfel T, Hoffmann-Sommergruber K, Roberts G, Beyer K, Bindslev-Jensen C (2014). EAACI food allergy and anaphylaxis guidelines: diagnosis and management of food allergy. Allergy.

[4] Burks AW, Tang M, Sicherer S, Muraro A, Eigenmann PA, Ebisawa M (2012). ICON: food allergy. J Allergy Clin Immunol.

[5] Sampson HA, Aceves S, Bock SA, James J, Jones S, Lang D (2014). Practice parameter workgroup. Food allergy: a practice parameter update-2014. J Allergy Clin Immunol.

[6] Nowak-Węgrzyn A, Chehade M, Groetch ME, Spergel JM, Wood RA, Allen K (2017). International consensus guidelines for the diagnosis and management of food protein-induced enterocolitis syndrome: executive summary-workgroup report of the adverse reactions to foods committee, American Academy of Allergy, Asthma & Immunology. J Allergy Clin Immunol.

[7] Reese I, Holzhauser T, Schnadt S, Dölle S, Kleine-Tebbe J, Raithel M (2015). Allergen and allergy risk assessment, allergen management, and gaps in the European food information regulation (FIR): are allergic consumers adequately protected by current statutory food safety and labeling regulations?. Allergo J Int.

[8] Dunn Galvin A, Chan CH, Crevel R, Grimshaw K, Poms R, Schnadt S (2015). Precautionary allergen labelling: perspectives from key stakeholder groups. Allergy.

[9] Giovannini M, D'Auria E, Caffarelli C, Verduci E, Barberi S, Indinnimeo L (2014). Nutritional management and follow up of infants and children with food allergy: Italian Society of Pediatric Nutrition/Italian Society of Pediatric Allergy and Immunology Task Force Position Statement. Ital J Pediatr.

[10] Gupta RS, Springston EE, Warrier MR, Smith B, Kumar R, Pongracic J (2011). The prevalence, severity, and distribution of childhood food allergy in the United States. Pediatrics.

[11] Chafen JJ, Newberry SJ, Riedl MA, Bravata DM, Maglione M, Suttorp MJ (2010). Diagnosing and managing common food allergies: a systematic review. JAMA.

[12] Savage J, Johns CB (2015). Food allergy: epidemiology and natural history. Immunol Allergy Clin N Am.

[13] Fiocchi A, Pecora V, Valluzzi RL, Fierro V, Mennini M (2017). Use of biologics in severe food allergies. Curr Opin Allergy Clin Immunol.

[14] Schofield AT (1908). A case of egg poisoning. Lancet.

[15] Potemkina AM, Timerbaeva GM (1982). Specific desensitization treatment of children with food hypersensitivity by the sublingual method. Pediatriia.

[16] Patriarca C, Romano A, Venuti A, Schiavino D, Di Rienzo V, Nucera E (1984). Oral specific hyposensitization in the management of patients allergic to food. Allergol Immunopathol (Madr).

[17] Casimir G, Cuvelier P, Allard S, Duchateau J (1997). Life-threatening fish allergy successfully treated with immunotherapy. Pediatr Allergy Immunol.

[18] Nelson HS, Lahr J, Rule R, Bock A, Leung D (1997). Treatment of anaphylactic sensitivity to peanuts by immunotherapy with injections of aqueous peanut extract. J Allergy Clin Immunol.

[19] Bauer A, Ekanayake Mudiyanselage S, Wigger-Alberti W, Elsner P (1999). Oral rush desensitization to milk. Allergy.

[20] Patriarca G, Nucera E, Pollastrini E, De Pasquale T, Lombardo C, Buonomo A (2006). Oral rush desensitization in peanut allergy: a case report. Dig Dis Sci.

[21] Patriarca G, Schiavino D, Nucera E, Schinco G, Milani A, Gasbarrini GB (1998). Food allergy in children: results of a standardized protocol for oral desensitization. Hepato-Gastroenterology.

[22] Meglio P, Bartone E, Plantamura M, Arabito E, Giampietro PG (2004). A protocol for oral desensitization in children with IgE mediated cow’s milk allergy. Allergy.

[23] Patriarca G, Nucera E, Roncallo C, Pollastrini E, Bartolozzi F, De Pasquale T (2003). Oral desensitizing treatment in food allergy: clinical and immunological results. Aliment Pharmacol Ther.

[24] Mansfield L (2006). Successful oral desensitization for systemic peanut allergy. Ann Allergy Asthma Immunol.

[25] Fisher HR, du Toit G, Lack G (2011). Specific oral tolerance induction in food allergic children: is oral desensitisation more effective than allergen avoidance?: a meta-analysis of published RCTs. Arch Dis Child.

[26] Yeung JP, Kloda LA, McDevitt J, Ben-Shoshan M, Alizadehfar R (2012). Oral immunotherapy for milk allergy. Cochrane Database Syst Rev.

[27] Brożek JL, Terracciano L, Hsu J, Kreis J, Compalati E, Santesso N (2012). Oral immunotherapy for IgE-mediated cow's milk allergy: a systematic review and meta-analysis. Clin Exp Allergy.

[28] Sun J, Hui X, Ying W, Liu D, Wang X (2014). Efficacy of allergen-specific immunotherapy for peanut allergy: a meta-analysis of randomized controlled trials. Allergy Asthma Proc.

[29] Romantsik O, Bruschettini M, Tosca MA, Zappettini S, Della Casa Alberighi O, Calevo MG (2014). Oral and sublingual immunotherapy for egg allergy. Cochrane Database Syst Rev.

[30] Meglio P, Giampietro PG, Gianni S, Galli E (2008). Oral desensitization in children with immunoglobulin E-mediated cow's milk allergy--follow-up at 4 yr and 8 months. Pediatr Allergy Immunol.

[31] Keet CA, Frischmeyer-Guerrerio PA, Thyagarajan A, Schroeder JT, Hamilton RG, Boden S (2012). The safety and efficacy of sublingual and oral immunotherapy for milk allergy. J Allergy Clin Immunol.

[32] Paassilta M, Salmivesi S, Mäki T, Helminen M, Korppi M (2016). Children who were treated with oral immunotherapy for cows' milk allergy showed long-term desensitisation seven years later. Acta Paediatr.

[33] Elizur A, Appel MY, Goldberg MR, Yichie T, Levy MB, Nachshon L (2016). Clinical and laboratory 2-year outcome of oral immunotherapy in patients with cow's milk allergy. Allergy.

[34] Berin MC, Sampson HA (2013). Mucosal immunology of food allergy. Curr Biol.

[35] Klebanoff CA, Spencer SP, Torabi-Parizi P, Grainger JR, Roychoudhuri R, Ji Y (2013). Retinoic acid controls the homeostasis of precDC- derived splenic and intestinal dendritic cells. J Exp Med.

[36] Coombes JL, Siddiqui KR, Arancibia-Carcamo CV, Hall J, Sun CM, Belkaid Y (2007). A functionally specialized population of mucosal CD103+ DCs induces Foxp3+ regulatory T cells via a TGF-beta and retinoic acid dependent mechanism. J Exp Med.

[37] Macia L, Tan J, Vieira AT, Leach K, Stanley D, Luong S (2015). Metabolite-sensing receptors GPR43 and GPR109A facilitate dietary fibre-induced gut homeostasis through regulation of the inflammasome. Nat Commun.

[38] Zaki MH, Boyd KL, Vogel P, Kastan MB, Lamkanfi M, Kanneganti TD (2010). The NLRP3 inflammasome protects against loss of epithelial integrity and mortality during experimental colitis. Immunity.

[39] Elinav E, Strowig T, Kau AL, Henao-Mejia J, Thaiss CA, Booth CJ (2011). NLRP6 inflammasome regulates colonic microbial ecology and risk for colitis. Cell.

[40] Akdis M, Akdis CA (2014). Mechanisms of allergen-specific immunotherapy: multiple suppressor factors at work in immune tolerance to allergens. J Allergy Clin Immunol.

[41] Dioszeghy V, Mondoulet L, Puteaux E, Dhelft V, Ligouis M, Plaquet C, et al. Differences in phenotype, homing properties and suppressive activities of regulatory T cells induced by epicutaneous, oral or sublingual immunotherapy in mice sensitized to peanut. Cell Mol Immunol. 2016 Apr 11; 10.1038/cmi.2016.14. [Epub ahead of print]. 10.1038/cmi.2016.14 PMC5596241 27063469

[42] Rifkin DB (2005). Latent transforming growth factor-beta (TGF-beta) binding proteins: orchestrators of TGF-beta availability. J Biol Chem.

[43] Gandhi R, Farez MF, Wang Y, Kozoriz D, Quintana FJ, Weiner HL (2010). Cutting edge: human latency-associated peptide+ T cells: a novel regulatory T cell subset. J Immunol.

[44] Leonard SA, Martos G, Wang W, Nowak-Węgrzyn A, Berin MC (2012). Oral immunotherapy induces local protective mechanisms in the gastrointestinal mucosa. J Allergy Clin Immunol.

[45] Rolinck-Werninghaus C, Staden U, Mehl A, Hamelmann E, Beyer K, Niggemann B (2005). Specific oral tolerance induction with food in children: transient or persistent effect on food allergy?. Allergy.

[46] Keet CA, Frischmeyer-Guerrerio PA, Thyagarajan A, Schroeder JT, Hamilton RG, Boden S (2012). The safety and efficacy of sublingual and oral immunotherapy for milk allergy. J Allergy Clin Immunol.

[47] Gorelik M, Narisety SD, Guerrerio AL, Chichester KL, Keet CA, Bieneman AP (2015). Suppression of the immunologic response to peanut during immunotherapy is often transient. J Allergy Clin Immunol.

[48] Burks AW, Jones SM, Wood RA, Fleischer DM, Sicherer SH, Lindblad RW (2012). Oral immunotherapy for treatment of egg allergy in children. N Engl J Med.

[49] Escudero C, Rodríguez Del Río P, Sánchez-García S, Pérez-Rangel I, Pérez-Farinós N, García-Fernández C (2015). Early sustained unresponsiveness after short-course egg oral immunotherapy: a randomized controlled study in egg-allergic children. Clin Exp Allergy.

[50] Yanagida N, Sato S, Asaumi T, Nagakura K, Ogura K, Ebisawa M (2016). Safety and efficacy of low-dose oral immunotherapy for Hen's egg allergy in children. Int Arch Allergy Immunol.

[51] Jones SM, Burks AW, Keet C, Vickery BP, Scurlock AM, Wood RA (2016). Long-term treatment with egg oral immunotherapy enhances sustained unresponsiveness that persists after cessation of therapy. J Allergy Clin Immunol.

[52] Yanagida N, Sato S, Asaumi T, Okada Y, Ogura K, Ebisawa M (2015). A single-center, case-control study of low-dose-induction oral immunotherapy with Cow's milk. Int Arch Allergy Immunol.

[53] Wood RA, Kim JS, Lindblad R, Nadeau K, Henning AK, Dawson P (2016). A randomized, double-blind, placebo-controlled study of omalizumab combined with oral immunotherapy for the treatment of cow's milk allergy. J Allergy Clin Immunol.

[54] Takahashi M, Taniuchi S, Soejima K, Hatano Y, Yamanouchi S, Kaneko K (2016). Two-weeks-sustained unresponsiveness by oral immunotherapy using microwave heated cow's milk for children with cow's milk allergy. Allergy Asthma Clin Immunol.

[55] Frischmeyer-Guerrerio PA, Masilamani M, Gu W, Brittain E, Wood R, Kim J, et al. Mechanistic correlates of clinical responses to omalizumab in the setting of oral immunotherapy for milk allergy. J Allergy Clin Immunol. 2017;140(4):1043-1053.e8. 10.1016/j.jaci.2017.03.028 PMC5632581 28414061

[56] Fiocchi A, Burks W, Bahna SL, Bielory L, Boyle RJ, Cocco R (2012). WAO special committee on food allergy and nutrition. Clinical use of probiotics in pediatric allergy (CUPPA): a world allergy organization position paper. World Allergy Organ J.

[57] Fiocchi A, Terracciano L, Bouygue GR, Veglia F, Sarratud T, Martelli A (2008). Incremental prognostic factors associated with cow's milk allergy outcomes in infant and child referrals: the Milan Cow's Milk Allergy Cohort study. Ann Allergy Asthma Immunol.

[58] Ho MH, Wong WH, Heine RG, Hosking CS, Hill DJ, Allen KJ (2008). Early clinical predictors of remission of peanut allergy in children. J Allergy Clin Immunol.

[59] Karlsson MR, Rugtveit J, Brandtzaeg P (2004). Allergen-responsive CD4+CD25+ regulatory T cells in children who have outgrown cow’s milk allergy. J Exp Med.

[60] Shreffler WG, Wanich N, Moloney M, Nowak-Wegrzyn A, Sampson HA (2009). Association of allergen-specific regulatory T cells with the onset of clinical tolerance to milk protein. J Allergy Clin Immunol.

